# Natural drivers of multidecadal Arctic sea ice variability over the last millennium

**DOI:** 10.1038/s41598-020-57472-2

**Published:** 2020-01-20

**Authors:** Paul R. Halloran, Ian R. Hall, Matthew Menary, David J. Reynolds, James D. Scourse, James A. Screen, Alessio Bozzo, Nick Dunstone, Steven Phipps, Andrew P. Schurer, Tetsuo Sueyoshi, Tianjun Zhou, Freya Garry

**Affiliations:** 1 College of Life and Environmental Sciences, University of Exeter, Exeter, UK; 2 School of Earth and Ocean Science, Cardiff University, Cardiff, CF10 3AT UK; 3 LOCEAN/IPSL, Sorbonne Universités (SU)‐CNRS‐IRD‐MNHN, Paris, France; 4 Laboratory of Tree Ring Research, The University of Arizona, Arizona, USA; 5 College of Life and Environmental Sciences, University of Exeter, Penryn Campus, Treliever Road, Penryn, Cornwall UK; 6 College of Engineering, Mathematics and Physical Sciences, University of Exeter, Exeter, UK; 7 Eumetsat, Darmstadt, Germany; 8 Met Office Hadley Centre, FitzRoy Road, Exeter, EX1 3PB UK; 9 Institute for Marine and Antarctic Studies, University of Tasmania, Private Bag 129, Hobart, TAS 7001 Australia; 10 School of Geosciences, The University of Edinburgh, Edinburgh, UK; 11 National Institute of Polar Research, 10-3 Midori-cho, Tachikawa-city, 190-8518 Japan; 12 Japan Agency for Marine-Earth Science and Technology, 3173-25, Showa-machi Kanazawa-ku, Yokohama City, 236-0001 Japan; 13 LASG, Institute of Atmospheric Physics, Chinese Academy of Sciences, Beijing, 100029 China

**Keywords:** Palaeoceanography, Climate and Earth system modelling, Cryospheric science

## Abstract

The climate varies due to human activity, natural climate cycles, and natural events external to the climate system. Understanding the different roles played by these drivers of variability is fundamental to predicting near-term climate change and changing extremes, and to attributing observed change to anthropogenic or natural factors. Natural drivers such as large explosive volcanic eruptions or multidecadal cycles in ocean circulation occur infrequently and are therefore poorly represented within the observational record. Here we turn to the first high-latitude annually-resolved and absolutely dated marine record spanning the last millennium, and the Paleoclimate Modelling Intercomparison Project (PMIP) Phase 3 Last Millennium climate model ensemble spanning the same time period, to examine the influence of natural climate drivers on Arctic sea ice. We show that bivalve oxygen isotope data are recording multidecadal Arctic sea ice variability and through the climate model ensemble demonstrate that external natural drivers explain up to third of this variability. Natural external forcing causes changes in sea-ice mediated export of freshwater into areas of active deep convection, affecting the strength of the Atlantic Meridional Overturning Circulation (AMOC) and thereby northward heat transport to the Arctic. This in turn leads to sustained anomalies in sea ice extent. The models capture these positive feedbacks, giving us improved confidence in their ability to simulate future sea ice in in a rapidly evolving Arctic.

## Introduction

Sea ice is one of the most dynamic components of our climate system. Changes in sea ice amplify energy imbalance in the polar regions, and may have significant impacts on mid-latitude weather and extremes^1,2^. The Arctic sea ice decline observed in recent decades, while robustly linked to anthropogenic forcing^3^, includes a contribution from internal variability^4^ and volcanic activity^5^. While the short-term response of sea ice to volcanic forcing can be understood in terms of reduction of incoming radiation, atmospheric reorganisation and changes in surface ocean temperature^6^, which can be examined in the observational record^7,8^, the multidecadal to centennial response is likely to be mediated through large-scale ocean dynamics such as the inflow of warm water to the Arctic within the upper limb of the AMOC^9–12^. Modelling studies have proposed a number of mechanisms through which volcanic activity can lead to decadal and longer changes in AMOC strength^13–15^, but agreement about how ocean circulation has changed on these timescales remains elusive. Understanding Arctic sea ice variability on multidecadal timescales therefore not only helps to better explain recently observed changes and potential predictability, but also to uncover the mechanisms of multidecadal variability in the wider climate system.

## Oxygen Isotope Variability Recorded from North Of Iceland

**Figure 1:**
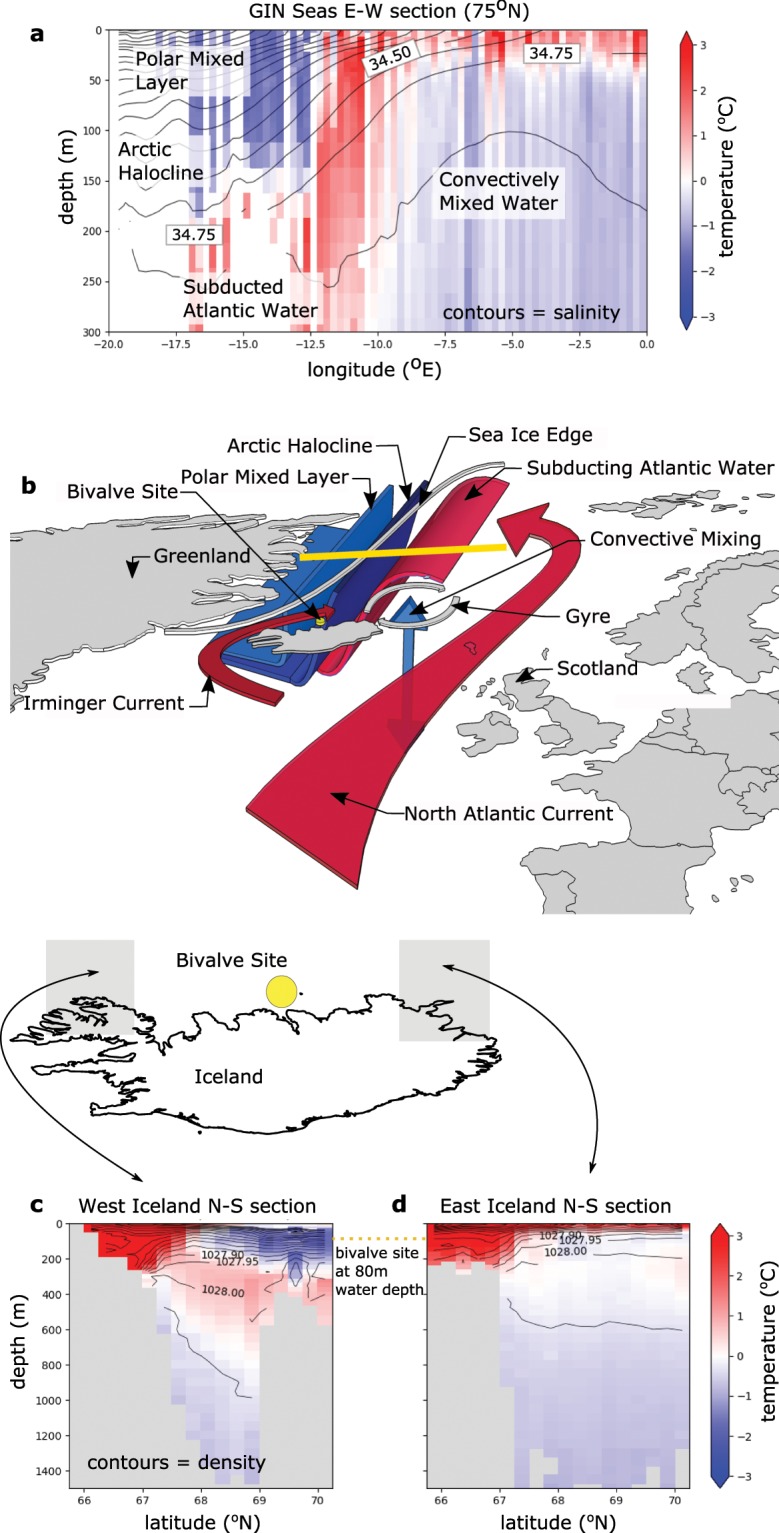
Oceanographic context. (**a**) Annual mean climatological (1955–2012 mean) temperature (colours) and salinity (contours) from a vertical section across 75°N (highlighted by the yellow bar), highlighting the different water-masses^22^. 75°N is chosen because it most clearly illustrated the water-mass structure. This water-mass structure is present throughout the open water of the GIN seas, but with the volume of Arctic-sources waters decreasing to the south. (**b**) Greenland-Iceland-Norway Seas schematic with bivalve site location (yellow circle), at 66°31.59′N, 18°11.74′W, 80 m water depth). c and d: Zonally averaged annual mean climatological (1955–2012 mean) temperature (contour colours, °C) and density (contour lines, kg.m^−3^) from the (**c**) West (averaged from 15.75–13.75°W) of Iceland and (**d**) East (averaged from 23.75–21.75°W) of Iceland^22^ as illustrated by the grey regions on the outline map of Iceland.

The Greenland-Iceland-Norway (GIN) Seas are characterised by a number of stratified water-masses with contrasting temperature and salinity properties. The position of these water masses is tightly coupled to that of the sea ice edge (Fig. 1b). The large area of land that drains into the Arctic, the net precipitative regime over the basin, and relatively fresh inflow through the Bering Strait results in a stratified low-salinity layer approximately 200 m thick sitting directly below the sea ice across much of the Arctic^16^. This low-salinity layer is made up of very fresh Polar Mixed Layer waters at the surface and the more saline Arctic Halocline waters below^16^ (Fig. 1a,b). Warm relatively saline waters enter the Arctic from the North Atlantic Current^17^, representing the upper limb of the AMOC (Fig. 1b). Where the North Atlantic Current waters meet the cold fresh waters, they are either entrained into the GIN Seas gyres, lose heat to the atmosphere and convectively mix with deeper Arctic waters^18^, or subduct beneath the fresher Arctic water-masses, where they fill much of the Arctic basin between about 200 and 800m below the surface^17^ (Fig. 1a,b). Within the region bounded by the winter sea ice edge, the Arctic Halocline waters are too fresh to mix convectively with the waters below^16^. The winter sea ice edge therefore marks the region where Atlantic waters subduct below the Halocline waters and, seaward of which, convection occurs (Fig. 1b).

**Figure 2:**
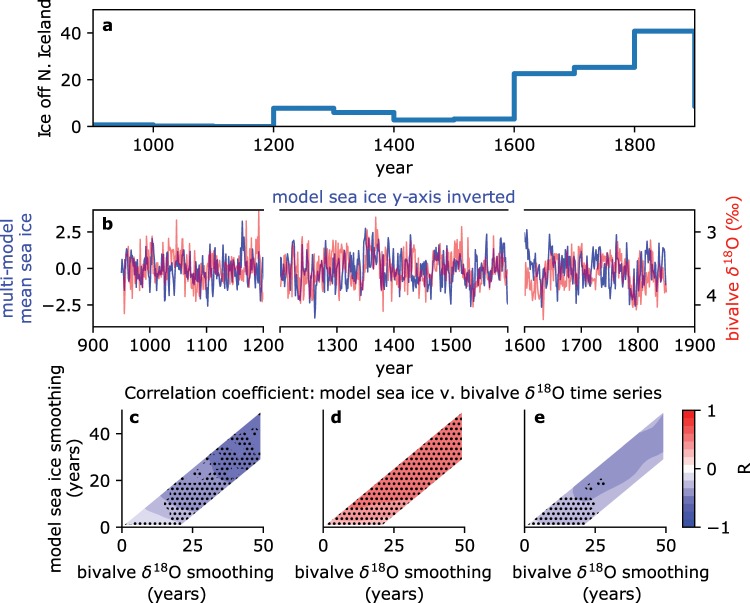
Correspondence of δ^18^O record and modelled Greenland-Iceland-Norway seas sea ice fraction. (**a**) Severity of ice off North Iceland coast calculated from documentary evidence of the duration of ice (total weeks) multiplied by the extent along the coast^27^. (**b**) Area-averaged PMIP3 multi-model mean Greenland-Iceland-Norward seas sea ice fraction anomaly (blue) and bivalve Arctica islandica δ^18^O record (red), both timeseries are linearly detrended and sea ice fraction is normalised by its standard deviation. Between 1200 CE and 1600 CE the model sea ice timeseries is inverted (multiplied by −1). Note that following convention, the bivalve δ^18^O axis is inverted so that warming is represented by a positive change. Lower Panels: Correlation coefficients between the sea ice and δ^18^O timeseries for the intervals 950–1200 CE (**c**), 1200–1600 CE (**d**) and 1600–1849 CE (**e**) presented across a range of smoothing windows (see Methods). Stippling highlights significance (see Methods). Correlation coefficients are presented with smoothing windows of up to 20 years away from the 1:1 line to accommodate smoothing already introduced by calculating the multi-model mean. Maximum positive or negative correlations are −0.58, +0.58 and −0.34 for the intervals shown in panels c, d and e respectively, leading to a maximum variance explained in any phase (R^2^) of 34%.

The North Iceland Shelf, although it remains in communication with the subpolar North Atlantic at the surface via the ﻿Irminger Current^18^ (Fig. 1b), in the subsurface transects the water-bodies of the GIN Seas (Fig. 1c,d). The western edge of the North Iceland Shelf is connected along isopycnal surfaces with the Arctic Halocline and Polar Mixed Layer waters near the surface, and the subducted Atlantic waters near the shelf edge (Fig. 1c). In contrast, the eastern edge of the North Iceland Shelf is connected along isopycnal surfaces to the convectively mixed Atlantic/deep-Arctic waters (Fig. 1d). In the centre of this transect an annually-resolved stable oxygen isotope (δ^18^O) record has been constructed spanning the last millennium from aragonite samples drilled from shell growth bands belonging to an *Arctica islandica* bivalve chronology^19,20^ (site location: 66°31.59′N, 18°11.74′W, 80 m water depth). The North Iceland bivalve δ^18^O timeseries, which is derived from aragonite precipitated in apparent equilibrium with the ambient seawater salinity and temperature^21^, displays marked multidecadal variability (Fig. 2b). Reynolds *et al*.^20^, take advantage of the annual-resolution and absolute dating in this record to identify the relative timing of change occurring within the ocean and that occurring on land, as a way to ask whether the ocean is passively responding to atmospheric change over this interval, or whether ocean change led atmospheric change and therefore played an active role in the key climate transitions of the last millennium. These authors found that the change recorded in the bivalve timeseries tends to precede Northern Hemisphere air-temperature change, and therefore suggested that the ocean played an important role in driving atmospheric change. However, the specific drivers of the ocean change remain unresolved. While solar irradiance and volcanic aerosol forcing have been linked to the high-frequency variability in the bivalve δ^18^O timeseries, no direct coherent multidecadal relationship is found between the record and natural external forcing^20^. Here we propose that the seawater δ^18^O recorded over time in bivalve shells from the North Iceland Shelf is faithfully documenting change in the relative influence of the different GIN Seas water masses at the site, as the sea ice extent varies in response to the integration and mediation of the external forcing by coupled climate system processes.

**Figure 3:**
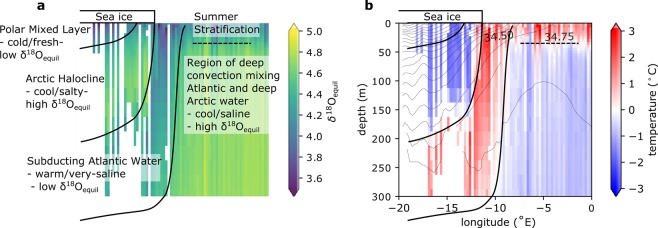
Relationship of oceanography, temperature and salinity to δ^18^O_equil._. δ^18^O_equil._ is defined here as the δ^18^O of aragonite in equilibrium with seawater at observed temperatures and salinities. (**a**) Calculated δ^18^O_equil._ climatology from climatological temperature and salinity values^22^ from vertical section across the GIN Seas at 75°N (see Methods), with water-masses annotated. (**b**) Temperature (contour colours) and salinity (contour lines) from vertical section across 75°N within the GIN Seas Regional Climatology^22^. The solid black lines depict the boundaries between water masses. The dashed black lines highlight where the water stratifies in summer, leading seasonally to the warm surface layer evident in panel b.

The expected isotopic composition of the bivalve aragonite can be predicted from thermodynamic equilibrium with the isotopic composition of the ambient water (δ^18^O_water_) at a given temperature, according to the empirical equation of Grossman and Ku^21^. Four distinct water-masses can be seen in δ^18^O_water_-space across the East-West GIN Seas transect, mapped out by calculating the δ^18^O of aragonite in equilibrium with seawater at observed temperatures and salinities^22^, described herein as the δ^18^O_equil._ (Fig. 3a) (see Methods).

## External Forcing of Sea Ice Extent

Observations and modelling studies have shown that sea ice perturbations can extend beyond the period over which the external forcing was present^9,11^. This means that sequential forcing events can interfere with each other, obscuring the relationship between the forcing and the resultant sea ice timeseries. The complexity of the sea ice response to external forcing necessitates the use of climate models to aid interpretation of records of past sea ice change. Phase three of the Paleoclimate Model Intercomparison Project (PMIP3)^23^ included simulations of the last millennium, forced by time varying land-use and greenhouse gas concentrations, orbital parameter changes, solar irradiance variability and volcanic aerosol concentration change^24^. Radiative forcing over the pre-industrial component of the last millennium (850 Common Era (CE) to 1849 CE) was dominated by volcanic and solar activity^24^. We find remarkable agreement between the PMIP3 multi-model mean GIN-Seas sea ice extent (mean of eight simulations, see Methods), and the annually resolved North Iceland Shelf δ^18^O recorded in the bivalve shells (Fig. 2b). Significant correlations of −0.58, +0.58 and −0.34 are seen respectively in the early (950–1200 CE), middle (1200–1600 CE) and late (1600–1849 CE) intervals of the study period over multidecadal timescales (see Methods). The early and late intervals present negative correlations, i.e. greater sea ice extent corresponding with a lower bivalve δ^18^O value (Fig. 2c,e), and the middle interval presents a positive correlation (Fig. 2d).

**Figure 4:**
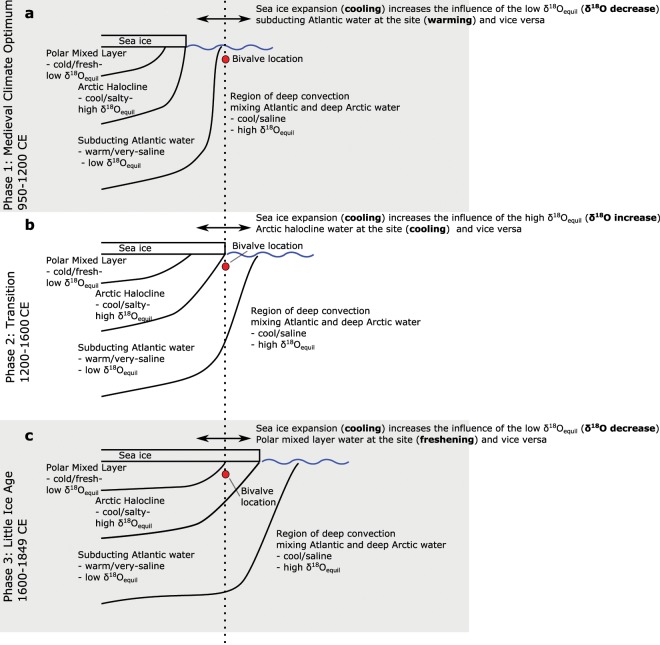
Diagrammatic explanation of the influence of variability in sea ice extent on δ^18^O_equil._ at the bivalve site. The relationship between the GIN Seas water-masses and the bivalve site under the three different approximate climatological sea ice states proposed for the three intervals highlighted in Fig. 2, and the implications for δ^18^O_equil._ variability in response to multidecadal (i.e. small-scale) sea ice variability around these three climatological states.

We propose that the three intervals identified in Fig. 2 correspond to three different hydrographic regimes where in the real ocean, but as discussed below not necessarily in models, multidecadal variability in the sea ice extent is superimposed on three different climatological sea ice states, and therefore water-mass geometries. The transition between regimes of Atlantic and Arctic water-mass influence at this site is supported by previous work^25^. The first interval coincides with the Medieval Climate Anomaly, where there is widespread evidence for anomalous warmth in the region surrounding the North Atlantic^26^, and little evidence of sea ice in the vicinity of the Icelandic coast^27^ (Fig. 2a). Within this interval, similar to the situation at the present day, we propose that the boundary between the cool and moderately saline high δ^18^O_eqil._ convectively-mixed waters and the warm and saline low δ^18^O_eqil._ subducting Atlantic waters was close to the bivalve site. In this state, multidecadal variability in sea ice extent, i.e. relatively small changes around the climatological state for that period, will have varied the influence of these two water-masses over the δ^18^O values recorded by the bivalves (Fig. 4a). Under these conditions an increase in sea ice extent would push the subducting Atlantic waters eastward and reduce the δ^18^O_eqil._ experienced at the bivalve site, and vice versa. Following a step increase in the documented incidence of sea ice off the coast of North Iceland around 1200 CE^27^ (Fig. 2a) we propose that the bivalve site was close to the boundary between the low δ^18^O_eqil._ subducting Atlantic waters and the cold and moderately saline Arctic Halocline waters. In this climatological state, multidecadal variability leading to an increase in sea ice extent would have increased the influence of low δ^18^O_eqil._ Arctic Halocline waters at the bivalve site (Fig. 4b), the opposite δ^18^O response than occurred during the preceding interval. The most recent interval (1600–1850 CE) follows a large increase in recorded sea ice off North Iceland^27^ (Fig. 2a) and corresponds to the expression of the Little Ice Age in the North Atlantic region^28^. During this most recent interval, we propose that the increased climatological sea ice extent would have moved the boundary between the high δ^18^O_eqil._ Arctic Halocline water and the cold and relatively fresh low δ^18^O_eqil._ Polar Mixed Layer Waters to the site (Fig. 4c). In its 1600–1850 CE configuration, a multidecadal sea ice expansion would result in an increased influence of the low δ^18^O_equil._ Polar Mixed Layer waters on the site, and again a negative δ^18^O versus sea ice relationship.

Correlation between the PMIP3 multi-model-mean sea ice and the bivalve δ^18^O timeseries reinforces our interpretation of the δ^18^O record as a proxy for multidecadal sea ice variability and suggest that the models are successful in representing the dynamical feedbacks allowing persistent sea ice change. The correlation also indicates that a significant component of the multidecadal variability in sea ice extent is externally forced, with the maximum variance explained being 34% (Fig. 2c–e). The phase of internal variability within the individual model simulations will be independent from each other, and so will largely cancel when averaged together (Fig. S1). The remaining variability will typify the externally forced signal.

While the PMIP3 models appear to correctly simulate the multidecadal sea ice variability, they do not capture the shifts between positive and negative bivalve δ^18^O versus sea ice correlations (Fig. 2b). The shifts between correlation regimes appear to arise from the interaction of stepwise sea ice extent advances from the Medieval Climate Anomaly into the Little Ice Age (Fig. 2a) with the detailed horizontal water-mass structure of the GIN Seas (Figs. 1 and 3a). The relatively coarse resolution PMIP3 models do not capture the observed water-mass structure of the GIN Seas (Fig. S2) and therefore, would not be expected to capture the switch in sign of the correlation between bivalve δ^18^O and sea ice timeseries. The transport of heat and freshwater from the Arctic coast into the interior of the basin to develop and sustain its stratification and therefore watermass structure, occurs through eddies^17^. These fine-scale features will not be captured in low resolution model simulations.

## Physical Mechanism

**Figure 5:**
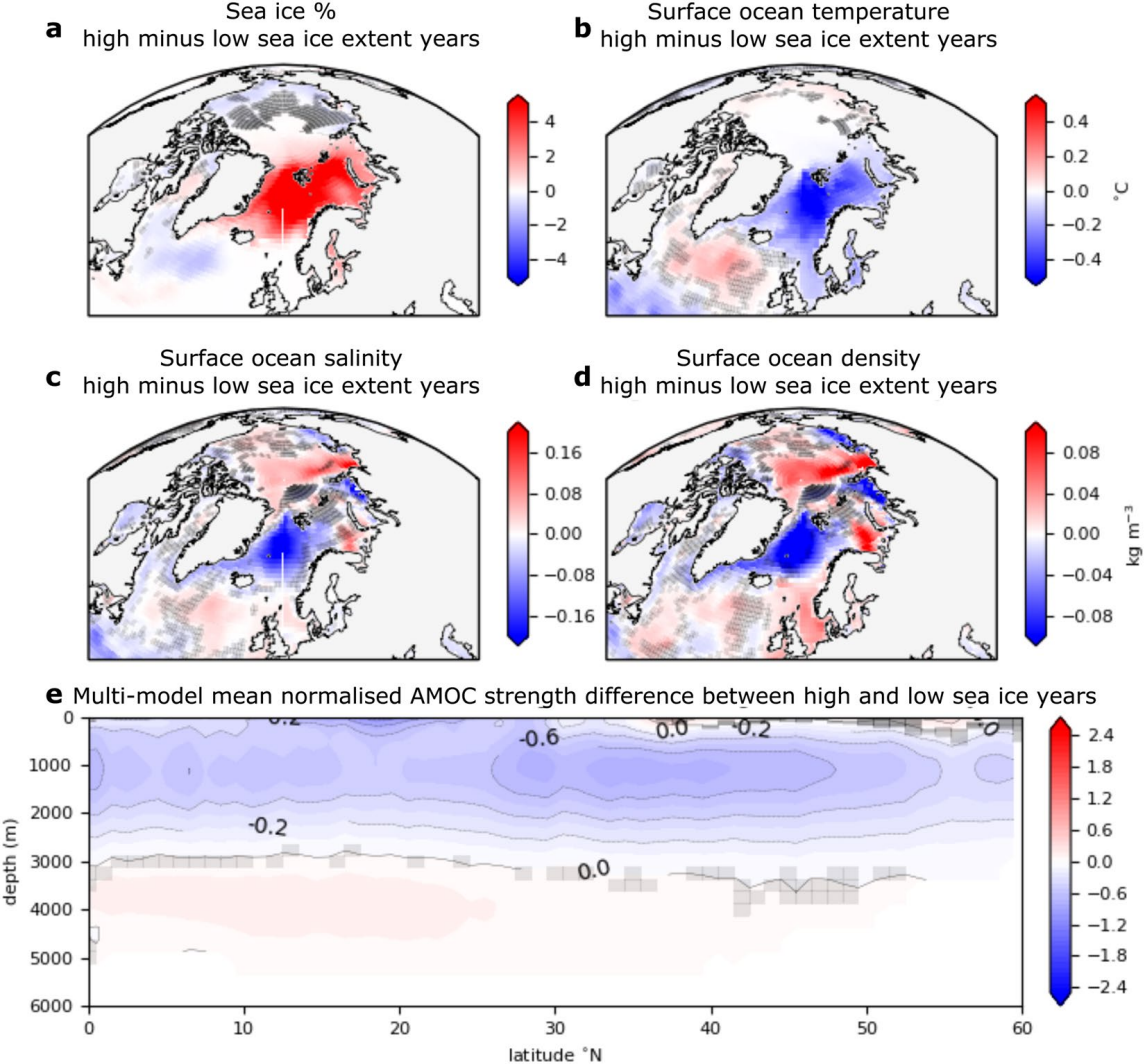
Proposed physical mechanism. Differences in sea ice fraction (**a**), surface ocean temperature (**b**), surface ocean salinity (**c**), surface ocean density (**d**) and normalised Atlantic Meridional Overturning stream function (**e**) composites from years with high and low sea ice extent within the multi-model mean sea ice timeseries, but excluding any years within the decade following a volcanic eruption (see Methods). Greyed out regions represent where fewer than 2/3^rds^ of the model agreed on the sign of change.

Multi-model-mean composites of Northern Hemisphere sea ice (see Methods) indicate that the variability constrained by the bivalve δ^18^O record is representative of change in the GIN, Barents and Kara Seas (Fig. 5a). Consistent with previous studies^9,11^ sea ice expansion coincides with a reduction in AMOC strength (Fig. 5e). The reduced heat transport to the Arctic via the AMOC, in response to both reduced AMOC strength and negative temperature anomalies, allows the expanded sea ice state to persist^9,11^, potentially reflecting a positive feedback between sea ice and the AMOC^9,11,29^. While there is substantial model evidence that natural external forcing drives AMOC variability^4,15,30,31^, the mechanisms and timescales of variability proposed by these studies differ^13^. Here multidecadal sea ice variability provides a proxy for AMOC variability, and agreement between modelled and reconstructed sea ice variability indicates that the PMIP3 models capture the feedback sustaining the sea ice anomalies on multidecadal timescales. Within this ensemble, it appears that the persistence of enhanced sea ice after external volcanic or solar forcing occurs in response to an AMOC-driven Atlantic-Arctic sea surface temperature cooling (Fig. 5a,b,e). A latitudinally coherent AMOC reduction is likely to occur in response to a density-driven reduction in high-latitude convection. A reduction in density in the sinking regions of the GIN Seas, northern Irminger Sea and perhaps Labrador Sea (Fig. 5d) can be largely attributed to salinity reductions in these regions (Fig. 5c). The reduction of salinity in the regions of active convective mixing is likely to result from the import of freshwater supplied by melting sea ice^9,11^ coming from the central Arctic and Barents Sea (Fig. 5c). The mechanism described here aligns with the suggestion that the reduction in heat-transport to the GIN Seas is dominated by a freshwater driven AMOC reduction^29^ rather than a sea ice driven Subpolar Atlantic cooling^11^. In summary, we find that initial sea ice perturbations occurring in response to the volcanic and solar forcing prescribed within the PMIP3 models^24^ appear to be sustained through export of freshwater in the form of sea ice to regions of active deep convection. Reduced convection leads to reduced AMOC strength, and thereby, to reduced heat transport to the sea ice edge via the upper limb of the AMOC and ultimately to expansion of sea ice cover.

Accurate near-term prediction, attribution of climatic events, and quantification of weather extremes relies on the ability of climate models to simulate internally and externally-driven variability on a range of timescales. Here we have presented a marine proxy-tested model reconstruction of multidecadal Arctic sea ice variability over the last millennium and identified an important contribution of natural external forcing to this variability. We have shown that climate models are capable of capturing the timing of this variability, despite the forcing being integrated by, and the response arising through, complex coupled atmosphere, sea ice and ocean processes. These results give us confidence that models are capable of simulating the oceanographic feedbacks which are likely to occur in response to anthropogenically driven sea ice decline.

## Methods

### Calculation of seawater δ^18^O_equil_

Annually-averaged temperature and salinity data from the NOAA/NCEI GIN Seas Regional Climatology^22^ was converted to a synthetic δ^18^O (${{\rm{\delta }}}^{18}{O}_{{equil}}$) value using Eqs. 1 and 2:

1 $${}^{18}Os{w}_{equil}=0.55\ast S-18.98$$

where the slope and intercept values are those identified for the North Atlantic in LeGrande and Schmidt^32^

2 $${}^{18}O_{equil}=\frac{21.8-T}{4.69}+({}^{18}Os{w}_{equil}-0.27)$$

following Reynolds *et al*.^20^ and Grossman and Ku^21^. Where S is the salinity and T is temperature in °C.

### Calculation of multi-model mean Arctic sea ice

The first ensemble member of all PMIP3 last millennium model simulations with the required variables, and compliant netcdf files were used, other than that of HadCM3^33^, which was assumed to be superseded by the more recent Met Office Hadley Center model (HadGEM2-ES) simulations. The model output used was from CCSM4^34^, CSIRO-Mk3L-1-2^35^, GISS-E2-R^36^, HadGEM2-ES^37,38^, MIROC-ESM^39,40^, MPI-ESM-P^41^, MRI-CGCM3^42^ and bcc-csm1-1^43^.

Model output was interpolated onto a regular 1 by 1 degree grid using the Climate Data Operator remapbil function^44^, and the sea ice fraction extracted for the region 45W-25E, 0N-90N and between the years 950CE and 1850CE, and averaged from monthly to yearly means. The area-weighted average timeseries was calculated for each model, this was linearly detrended, then the multi-model mean timeseries calculated.

### Correlation between multi-model mean sea ice and bivalve δ^18^O timeseries

The correlation coefficient between the multi-model-mean sea ice timeseries and the bivalve δ^18^O timeseries presented in Fig. 2c–e is calculated under rolling boxcar smoothing windows ranging from 1 to 50 years. The correlation coefficient is calculated for the two timeseries under independent smoothing windows to accommodate the fact that the calculation of a multi-model-mean is itself smoothing the sea ice timeseries. Autocorrelation associated with smoothing of the timeseries means that it is not appropriate to assign significance from the *p*-value of the correlations. Significance is therefore defined here as where the correlation coefficient exceeds (or is more negative than, in the case of a negative correlation) the correlation coefficient of at least 95% of 100 randomly generated timeseries of the same length which have undergone the same smoothing. The synthetic timeseries were generated with a uniform distribution between 0 and 1. It should be noted that this significance test is not directly analogous to a *p*-value, and the precise outcome is conditional on the spectral characteristics of the synthetic timeseries. The timing of the partitioning of the modelled sea ice timeseries into three periods (Fig. 1b) was undertaken based upon the timing of step changes in the observation-based index of ice severity off North Iceland (Fig. 2a).

### Composite anomalies

The first ensemble member of all PMIP3 last millennium model simulations with the required variables, and compliant netcdf files were used, other than that of HadCM3^33^, which was assumed to be superseded by the more recent Met Office Hadley Center model (HadGEM2-ES) simulations. For sea ice and salinity analysis these models were CCSM4, CSIRO-Mk3L-1-2, HadGEM2-ES, MIROC-ESM, MPI-ESM-P, MRI-CGCM3, bcc-csm1-1, and for AMOC analysis (calculated from ocean meridional velocities) these were bcc-csm1-1, CCSM4, CSIRO-Mk3L-1-2, FGOALS-gl^45^, MIROC-ESM, MPI-ESM-P and HadGEM2-ES.

Model output was interpolated onto a regular 1 by 1 degree grid using the Climate Data Operator remapbil function^44^, and the calculated Atlantic stream function data interpolated onto common depth levels using the Python scipy RectBivariateSpline function. High and low sea ice years were defined as where the multi-model-mean sea ice timeseries (normalised by its standard deviation) was greater than 1 standard deviation and less than −1 standard deviation from the mean respectively, but excluding the 10 years following a volcanic eruption with a global total stratospheric sulfate aerosol injection greater than 15Tg^46^. A subset of each 3D (latitude-longitude-time, or for AMOC analysis, latitude-depth-time) model field for each variable was selected, containing data from only the high and low sea ice years respectively. These fields were averaged along the time dimension, and the difference for each model between the high and low sea ice composites calculated. The multi-model mean was calculated from these composite difference fields.

## Supplementary information

Supplementary Information.
