# Supplementary material for: Natural drivers of multidecadal Arctic sea ice variability over the last millennium
Source: Sci Rep. 2020 Jan 20;10:688. doi: 10.1038/s41598-020-57472-2 (PMC6971300; doi:10.1038/s41598-020-57472-2)
Supplement: Supplementary file 1 — Supplementary Information. [file 41598_2020_57472_MOESM1_ESM.docx]

**Supplementary Information for Natural drivers of multidecadal Arctic sea ice variability over the last millennium**

Paul R. Halloran^1*^, Ian R. Hall^2^, Matthew Menary^3^, David J. Reynolds^2, 4^, James D. Scourse^5^, James A. Screen^6^, Alessio Bozzo^7^, Nick Dunstone^8^, Steven Phipps^9^, Andrew P. Schurer^10^, Tetsuo Sueyoshi^11,12^, Tianjun Zhou^13^, Freya Garry^1^.

**Affiliations**

^1^ College of Life and Environmental Sciences, University of Exeter, Exeter, UK
^2^ School of Earth and Ocean Science, Cardiff University, Cardiff, CF10 3AT, UK

^3^ LOCEAN/IPSL, Sorbonne Universités (SU)‐CNRS‐IRD‐MNHN, Paris, France

^4^ Laboratory of Tree Ring Research, The University of Arazona, USA

^5^ College of Life and Environmental Sciences, University of Exeter, Penryn Campus, Treliever Road, Penryn, Cornwall, UK

^6^ College of Engineering, Mathematics and Physical Sciences, University of Exeter, Exeter, UK

^7^ Eumetsat, Darmstadt, Germany

^8^ Met Office Hadley Centre, FitzRoy Road, Exeter EX1 3PB, UK

^9^ Institute for Marine and Antarctic Studies, University of Tasmania, Private Bag 129, Hobart, TAS 7001, Australia

^10^ School of Geosciences, The University of Edinburgh, Edinburgh, UK

^11^ National Institute of Polar Research, 10-3 Midori-cho, Tachikawa-city, 190-8518 Japan

^12^ Japan Agency for Marine-Earth Science and Technology, 3173-25, Showa-machi Kanazawa-ku, Yokohama City, 236-0001, Japan

^13^ LASG, Institute of Atmospheric Physics, Chinese Academy of Sciences, Beijing 100029, China

*Corresponding author: [p.halloran@exeter.ac.uk](mailto:p.halloran@exeter.ac.uk)


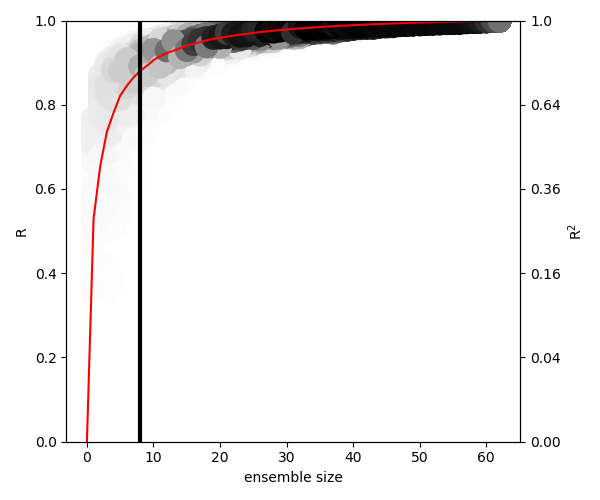


**Figure S 1. Atlantic sector mean Arctic sea ice fraction for ensemble member correlated with ensemble mean.** The mean of an infinitely large model ensemble would average out all internal variability generated by the models and only the forced signal would remain. The PMIP3 Last Millennium ensemble used in this study has 8 members, and as such not all internal variability will be removed by examining the multi-model mean. To assess how much of the forced signal is described by the mean of an eight-member ensemble the results presented in this figure turn to the much larger CMIP5 Historical-Nat ensemble, an experiment spanning the industrial era, but only including natural climate forcings ^55^, and therefore analogous to the past millennium simulations. The Historical-Nat ensemble contains 18 models for which sea ice concentration data is provided, and for each of these models, multiple simulations have been submitted with different initial conditions. Together an ensemble of 62 simulations is available. We construct 1000 randomly selected sub-ensembles from this 62 member ensemble for 60 different ensemble sizes (1 to 61 members). The correlation between the multi-model mean timeseries from each sub-ensemble with the multi-model mean timeseries from the full 62 member ensemble is plotted (grey transparent circular markers, getting darker as multiple markers plot on top of each other). The mean of the 1000 random sub-ensembles of each size is plotted in red. Convergence of the red line with an R-value of 1 well before the maximum ensemble size is reached indicates that the 62 member ensemble adequately approximates the forced signal. An ensemble size of 8, as used within the main study is likely to explain about 70% of the of the forced signal (black vertical line intersecting with red curve).


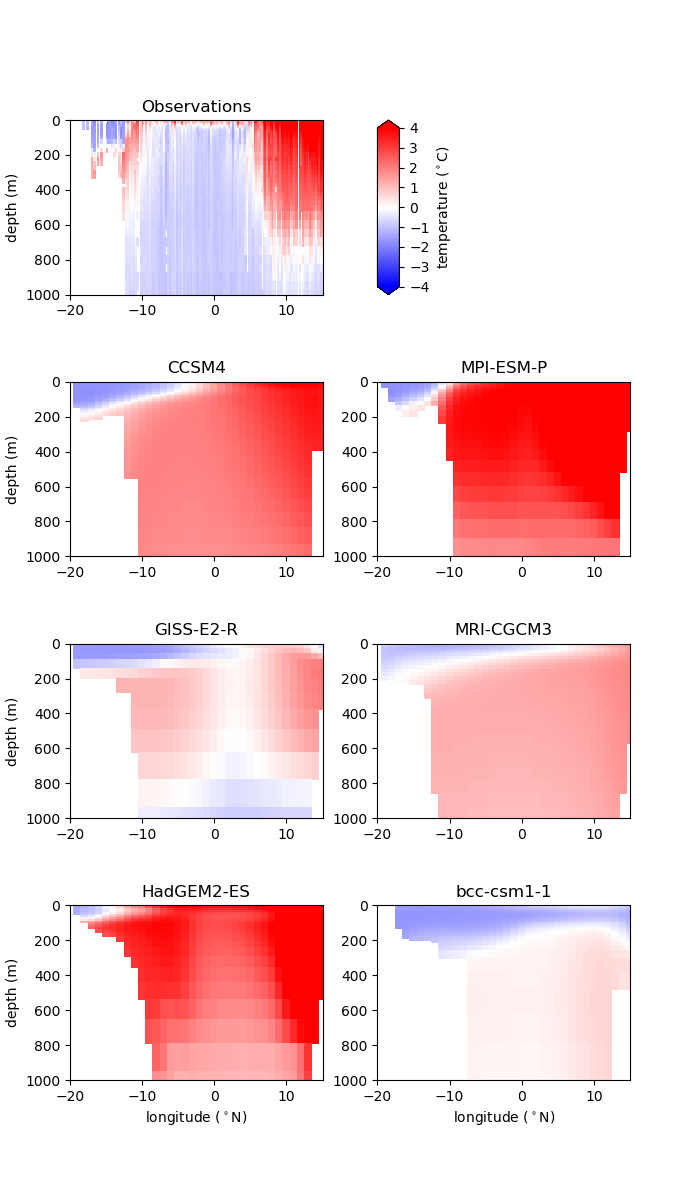


**Figure S 2. Model representation of GIN Seas oceanography.** Annual mean climatological (1955-2012) observed temperature from a vertical longitudinal transect across the GIN Seas at 75°N ^19^ (top), and equivalent plots calculated using PMIP3 model data from 1980-2000. A subset of the models used in the full study are presented due to data availability. While all models simulate the subduction of warm Atlantic water beneath the cooler Arctic water, and MPI-ESM-P, GISS-E2-R and HadGEM2-ES show evidence of convective mixing, in the form of penetration of slightly cooler waters at around 0°E towards the surface, no models show the clearly defined water-mass structure evident in the observations and required by the mechanism proposed to explain the shifts between positive and negative sea ice and bivalve δ^18^O relationships. Sea Surface Temperature and Salinity, and surface ocean seasonal sea ice extent maps place these results in a spatial context (Figure S3).


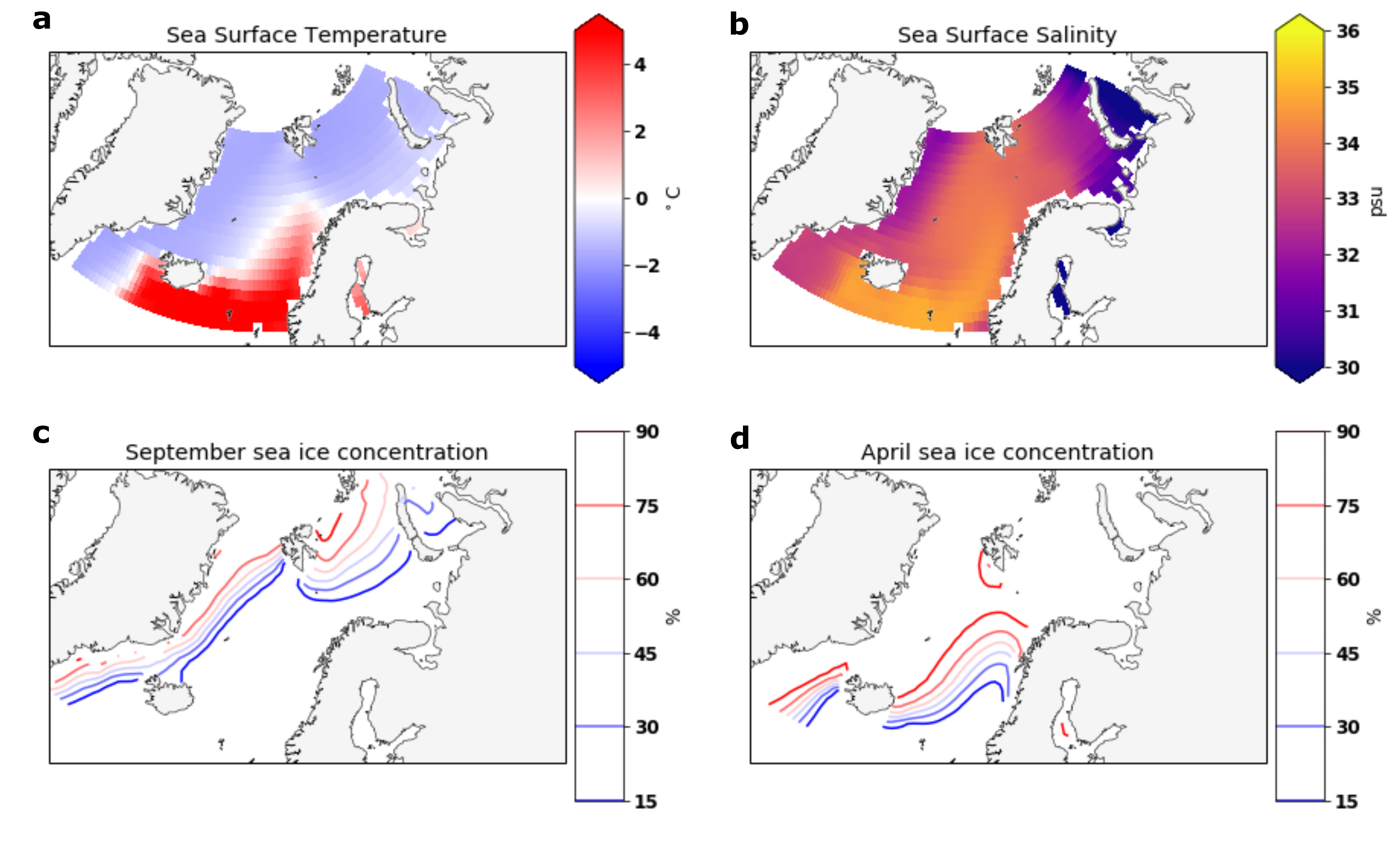


Figure S3 Multi-model mean climatological GIN Sea: Sea Surface Temperature (a), Sea Surface Temperature (b), September sea ice concentration (c) and April sea ice concentration (d). Climatologies are calculated from all years in the last millennium simulation.
